# Fast histological assessment of adipose tissue inflammation by label-free mid-infrared optoacoustic microscopy

**DOI:** 10.1038/s44303-023-00003-1

**Published:** 2023-12-06

**Authors:** Vito Ko, Marie C. Goess, Lukas Scheel-Platz, Tao Yuan, Andriy Chmyrov, Dominik Jüstel, Jürgen Ruland, Vasilis Ntziachristos, Selina J. Keppler, Miguel A. Pleitez

**Affiliations:** 1 Chair of Biological Imaging at the Central Institute for Translational Cancer Research (TranslaTUM), School of Medicine, Technical University of Munich, Munich, Germany; 2 Institute of Biological and Medical Imaging, Helmholtz Zentrum München, Neuherberg, Germany; 3 Institute for Clinical Chemistry and Pathobiochemistry, Technical University of Munich, School of Medicine, Munich, Germany; 4 TranslaTUM, Center for Translational Cancer Research, Technical University of Munich, Munich, Germany; 5 Institute of Computational Biology, Helmholtz Zentrum München, Neuherberg, Germany; 6 German Cancer Consortium (DKTK), Heidelberg, Germany; 7 German Center for Infection Research (DZIF), Munich, Germany; 8 Munich Institute of Biomedical Engineering (MIBE), Technical University of Munich, Garching b. München, Germany; 9 Division of Rheumatology and Clinical Immunology, Medical University Graz, Graz, Austria

**Keywords:** Biochemistry, Biotechnology, Chemistry, Analytical chemistry, Biochemistry, Applied optics, Optics and photonics, Optical techniques, Imaging and sensing, Microscopy, Optical spectroscopy, Immunology, Engineering, Biomedical engineering

## Abstract

Conventional histology, as well as immunohistochemistry or immunofluorescence, enables the study of morphological and phenotypical changes during tissue inflammation with single-cell accuracy. However, although highly specific, such techniques require multiple time-consuming steps to apply exogenous labels, which might result in morphological deviations from native tissue structures. Unlike these techniques, mid-infrared (mid-IR) microspectroscopy is a label-free optical imaging method that retrieves endogenous biomolecular contrast without altering the native composition of the samples. Nevertheless, due to the strong optical absorption of water in biological tissues, conventional mid-IR microspectroscopy has been limited to dried thin (5–10 µm) tissue preparations and, thus, it also requires time-consuming steps—comparable to conventional imaging techniques. Here, as a step towards label-free analytical histology of unprocessed tissues, we applied mid-IR optoacoustic microscopy (MiROM) to retrieve intrinsic molecular contrast by vibrational excitation and, simultaneously, to overcome water-tissue opacity of conventional mid-IR imaging in thick (mm range) tissues. In this proof-of-concept study, we demonstrated application of MiROM for the fast, label-free, non-destructive assessment of the hallmarks of inflammation in excised white adipose tissue; i.e., formation of crown-like structures and changes in adipocyte morphology.

## Introduction

The broad exposure to a “Western lifestyle” has led to a worldwide rise in mostly preventable diseases linked to chronic inflammation; for example, obesity-induced diabetes^1^ and inflammatory bowel disease (IBD)^2^. In this context, increasing evidence points to a link between white adipose tissue (WAT) and chronic inflammation. WAT controls energy homeostasis via the storage and release of lipids, but it can also influence inflammation by producing pro- and anti-inflammatory proteins. Under homeostatic conditions, WAT contains mainly a heterogeneous population of adipose-tissue macrophages (ATMs). Disturbed homeostasis in WAT leading to adipocyte death will tip the balance towards an inflammatory state, with concomitant phenotypic changes and a massive accumulation of ATMs followed by other immune cells^3^. Inflamed WAT is characterized by the formation of crown-like structures (CLS), consisting of macrophages surrounding dead or dying adipocytes, and changes in adipocyte morphology. These hallmarks of WAT inflammation can be assessed with high specificity by laser scanning confocal microscopy (LSCM) with the aid of immunohistochemistry or immunofluorescence^4–6^. However, tissue labeling is limited to the availability of antibodies and is a time-consuming procedure that requires precise a priori knowledge of the targeted molecules and complex laboratory steps, such as fixation, blocking, staining, sectioning, etc; which might introduce morphological deviations from native structures. Changes in the biomolecular composition in adipose tissue are difficult to address using immunohistochemistry-based labels and although mass spectrometry (MS) can be used as an alternative label-free analytical method, its implementation is even more time consuming. A better understanding of the native (unperturbed) metabolic and phenotypic changes in WAT during inflammation would be beneficial for the development of therapeutic strategies; however, analytical methods to access the native state of adipose tissue in the context of inflammation have been, so far, restrictive.

Unlike conventional histology or immunofluorescence, label-free optical imaging methods that retrieve endogenous molecular contrast from cells or tissue are less time-consuming and do not interfere with the native biomolecular composition of samples, thus allowing fast assessment of freshly excised WAT.

Nonlinear microscopy modalities such as second- and third- harmonic generation can be used to image some proteins (collagen/myosin) and lipids in cells and tissues. However, these methods detect optical contrast based on the spatial organization and optical heterogeneity of the imaged structures but not on their chemical composition^7–9^. Contrary to these methods, vibrational techniques, such as Raman- and mid-infrared-based imaging, are highly sensitive to detecting intrinsic biomolecular contrast by means of their characteristic vibrational modes. In particular, Coherent Anti-Stokes Raman scattering (CARS) and Stimulated Raman Scattering (SRS) microscopy have been extensively applied to visualize lipid distribution in WAT—mainly using the CH spectral region (3300–2700 cm^−1^) dominated by the vibrational modes of lipids^10–14^ but with limited sensitivity in the fingerprint region (1800–900 cm^−1^) characteristic of other important biomolecules such as carbohydrates and proteins^15^. Additionally, with the capability to image cross sections up to eight orders of magnitude larger than Raman imaging^16^, mid-infrared (mid-IR) spectroscopy promises to enhance sensitivity and specificity for label-free analytical histology even in the fingerprint region. For example, Fourier Transform Infrared (FTIR) microscopy was applied to image structural changes in WAT during obesity and trans-differentiation from brown to white adipocytes^17^. Nevertheless, the strong optical absorption of water renders freshly excised (unprocessed) biological tissues opaque to conventional mid-IR detection methods and, thus, most of the studies in WAT by mid-IR microspectroscopy have been limited to dry thin tissue preparations (5–10 µm) as in conventional imaging techniques.

To overcome the problem of tissue opacity, we previously developed Mid-infraRed Optoacoustic Microscopy (MiROM), which allows visualization of lipids and proteins in unprocessed tissues—recently applied for the histological characterization of carotid atherosclerosis^18,19^. Here, as a step towards label-free analytical histology of unprocessed tissues, we extended MiROM’s functionality to assess morphological hallmarks of inflamed WAT using mid-IR absorption bands as sources of intrinsic contrast instead of exogenous markers. Using MiROM here, we show the label-free identification of CLS and quantification of adipocyte sizes distributions—demonstrating MiROM’s potential as an imaging tool for fast, label-free, non-destructive assessment of the inflammatory state of unprocessed WAT.

## Results

**Fig. 1:**
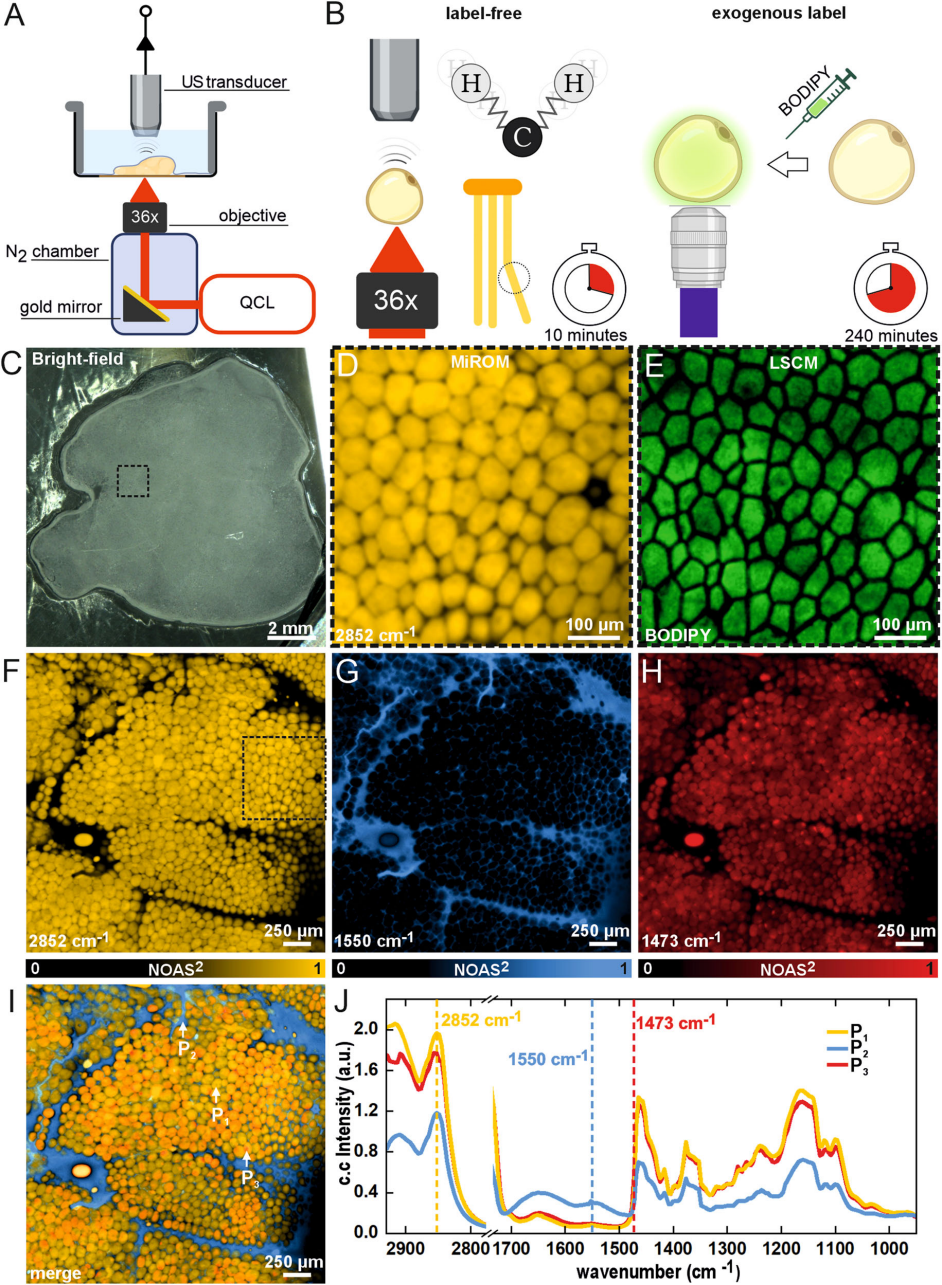
Mid-infrared optoacoustic microscopy (MiROM) for white adipose tissue (WAT) imaging. **A** Schematic setup of MiROM for WAT imaging. **B** MiROM uses the intrinsic contrast of lipids at 2852 cm^−1^ for label-free imaging of adipocytes instead of BODIPY-labeling, as in laser scanning confocal microscopy (LSCM), resulting in substantial reduction of sample preparation time. **C** WAT appears opaque in a bright field image (400–700 nm). **D**, **E** MiROM micrographs of WAT at 2852 cm^−1^ reveal intrinsic lipid content originating from adipocytes (**D**) similar to lipid content observed using BODIPY staining and LSCM imaging (**E**), imaged Field-of-view (FOV) marked in panel (**F**). **F**–**H** MiROM micrographs of the FOV marked in panel (**C**) for endogenous contrast at 2852 cm^−1^ (CH_2_ symmetric stretching), 1550 cm^−1^ (N-H bending/C-N stretching), and 1473 cm^−1^ (CH_2_/ CH_3_ bending/scissoring). FOV: 2 × 2 mm^2^ (cropped from 2 × 3 mm^2^), 5 μm step size. Imaging and tissue processing times in Table 1. **I** Three-channel micrograph showing three merged wavenumbers with three features of interest pointed to by arrows: normal adipocyte (P_1_), extracellular matrix (P_2_), and contrast accumulation at 1473 cm^−1^ (P_3_). **J** Spectra corresponding to the points marked in **I**. The excitation wavelengths of the micrograph panels in (**F**–**H**) are marked with vertical dashed lines with color corresponding to the micrographs. Illustrations **A**, **B** were created with BioRender.com.

Figure 1A schematically shows the excitation-detection configuration of MiROM for fresh tissue imaging. The details of MiROM’s principle, instrumentation, and imaging performance have been published elsewhere^18^. Briefly, MiROM accommodates co-aligned mid-IR excitation and ultrasound detection in the same focal plane. The tissue is illuminated with diffraction-limited focused mid-IR light (spot size ~5 µm, see Methods for details), provided by a broadly tunable quantum cascade laser (QCL), to generate optoacoustic (OA) signals by optical absorption. A MiROM image is obtained by raster-scanning the sample and retrieving the OA signal amplitude at each excited point at selected vibrational transitions. For instance, visualization of intrinsic lipid contrast in tissues is attainable via excitation of the symmetric stretching of CH_2_ molecules at 2852 cm^−1^, as shown by visualization of adipocytes primarily consisting of triglycerides (Fig. 1B), which are typically imaged by LSCM using BODIPY as a lipid-specific label (Fig. 1B). Generally, without the use of exogenous labels, WAT appears colorless and opaque when using (for instance) bright-field imaging in the visible region (400–700 nm), see Fig. 1C. In contrast, MiROM micrographs of WAT at 2852 cm^−1^ clearly revealed intrinsic lipid content of adipocytes (Fig. 1D), which matched the adipocyte distribution observed by LSCM using BODIPY staining for the same ROI as demonstrated in Fig. 1E (see also Supplementary Fig. 1A, B). Importantly, for the micrographs shown in Fig. 1, tissue preparation, including BODIPY staining, for LSCM required 4 h. In comparison, tissue preparation for MiROM imaging only required 10 min as staining is unnecessary. Thus, contrary to imaging with LSCM and BODIPY labeling, WAT imaging using MiROM required substantially less time during tissue preparation steps by avoiding the need for BODIPY labeling. This markedly shorter preparation time allows micrographs to be acquired faster compared to image acquisition using LSCM (i.e., total time of 1.7 h for MiROM compared to 4.15 h for LSCM) despite MiROM currently requiring a longer image acquisition time than a commercial LSCM (see Methods section and Table 1 for details).

**Table 1:** Comparison of imaging/process times for WAT tissues per channel/label.

Process	Fig. 1		Fig. 2	
	MiROM	LSCM (BODIPY)	MiROM	LSCM (BODIPY/MHCII)
Microscope setup	1 h	5 min	1 h	5 min
Sample preparation	10 min	3 h	10 min	3 h
Staining	0	1 h	0	24 h
Imaging	35 min	4 min	12 min	10 s
Acquired FOV	2 × 3 mm	3.7 × 3.2 mm	1 × 2 mm	581 × 581 um
Step size	5 µm	2048 nm (voxel size)	5 µm	2048 nm (voxel size)
Total time	1.75 h	4.15 h	1.37 h	27.08 h

Beyond single-contrast imaging, MiROM enables microscopic investigation at multiple vibrational transitions. This is demonstrated in Supplementary Fig. 2, where multiple sources of intrinsic contrast could be visualized by imaging WAT at eight different wavenumbers. In particular, Fig. 1F–H highlights micrographs at three selected wavenumbers: 2852, 1550, and 1473 cm^−1^ which are associated with CH_2_ symmetric stretching, N-H bending/C-N stretching, and CH_2_/ CH_3_ bending^20–23^, respectively. As mentioned above, contrast at 2852 cm^−1^ in Fig. 1F originated from lipid-rich adipocytes with a contrast-to-noise ratio (CNR) of 344:1 (see Methods for details). At 1550 cm^−1^ (Fig. 1G), the signal primarily originated from the amide II band of proteins constituting the extracellular matrix (ECM) and neighboring cells as well as from water content in the tissue. We observed up to 82:1 CNR for protein contrast in the areas surrounding the adipocytes, while protein contrast yielded less than 8:1 CNR inside adipocytes. The substantial difference in protein contrast between adipocytes and ECM—due to lower protein content and hydrophobicity of lipid droplets^24^—makes adipocytes appear opaque in 1550 cm^−1^ micrographs. Moreover, we found higher CNR values for lipid and protein contrast in freshly excised WAT; i.e., where no tissue fixation was applied (Supplementary Fig. 3A–D). Interestingly, at first sight, the contrast distribution for 1473 cm^−1^ (Fig. 1H) appeared similar to micrographs at 2852 cm^−1^. However, closer inspection revealed small features (roughly 10 times smaller than adipocytes) overlapping at the edge of adipocytes. These features are not visible at 2850 cm^−1^, but are observed in 1473 cm^−1^, see Supplementary Fig. 4. Additionally, a Structural Similarity (SSIM) analysis between both micrographs reported a SSIM index of 0.59—further supporting non negligible differences between the micrographs obtained at these two wavelengths. The OA contrast obtained at 1473 cm^−1^ shown in Fig. 1H might be representative of the absorption peak at 1463 cm^−1^, which is associated with CH_2_/CH_3_ bending^22,23^. Micrographs at 1473 cm^−1^ showed higher CNR (up to 135:1) compared to the CNR of micrographs at 2852 cm^−1^ from adipocytes (less than 106:1). Although not currently explicitly identified, we hypothesize that the localized contrast at 1473 cm^−1^ could potentially originate from lipid varieties with more dominant CH_2_/CH_3_ molecular bending response. Figure 1I and Supplementary Fig. 4 show merged-contrast micrographs in which the localized contrast forming the features at 1473 cm^−1^ accompanying the adipocytes are more clearly visible.

Additionally, MiROM enables spectral analysis at structures of interest identified in the micrographs. Mid-IR spectra were acquired by illuminating the selected structure with focused radiation to generate OA signal while tuning the QCL along the spectral range between 2932 and 910 cm^−1^ (see Methods for details). Figure 1J exemplifies the spectra from selected structures observed at three different points (P_1_, P_2_, and P_3_) in the micrographs in Fig. 1I. While no interfering spectral features from the fixing reagent were observed, see Supplementary Fig. 3E, the spectrum at P_1_ (adipocyte location) corresponds to the spectrum of triglycerides (as validated with FTIR measurements, see Supplementary Fig. 5). This is expected since lipid droplets in adipocytes are composed predominantly of triglycerides^25^. In the spectrum obtained from the adjacent ECM surrounding the adipocyte at P_2_, we observed a higher OA contrast attributed to high protein content. The spectrum obtained at P_3_ (representative of the red features in Fig. 1I) shows a similar profile as for triglycerides; although at 90% of adipocyte intensity (P_1_) at 2852 cm^−1^ and slightly higher intensity in the amide I and II regions (1.17-fold of adipocyte intensity in 1550 cm^−1^).

**Fig. 2:**
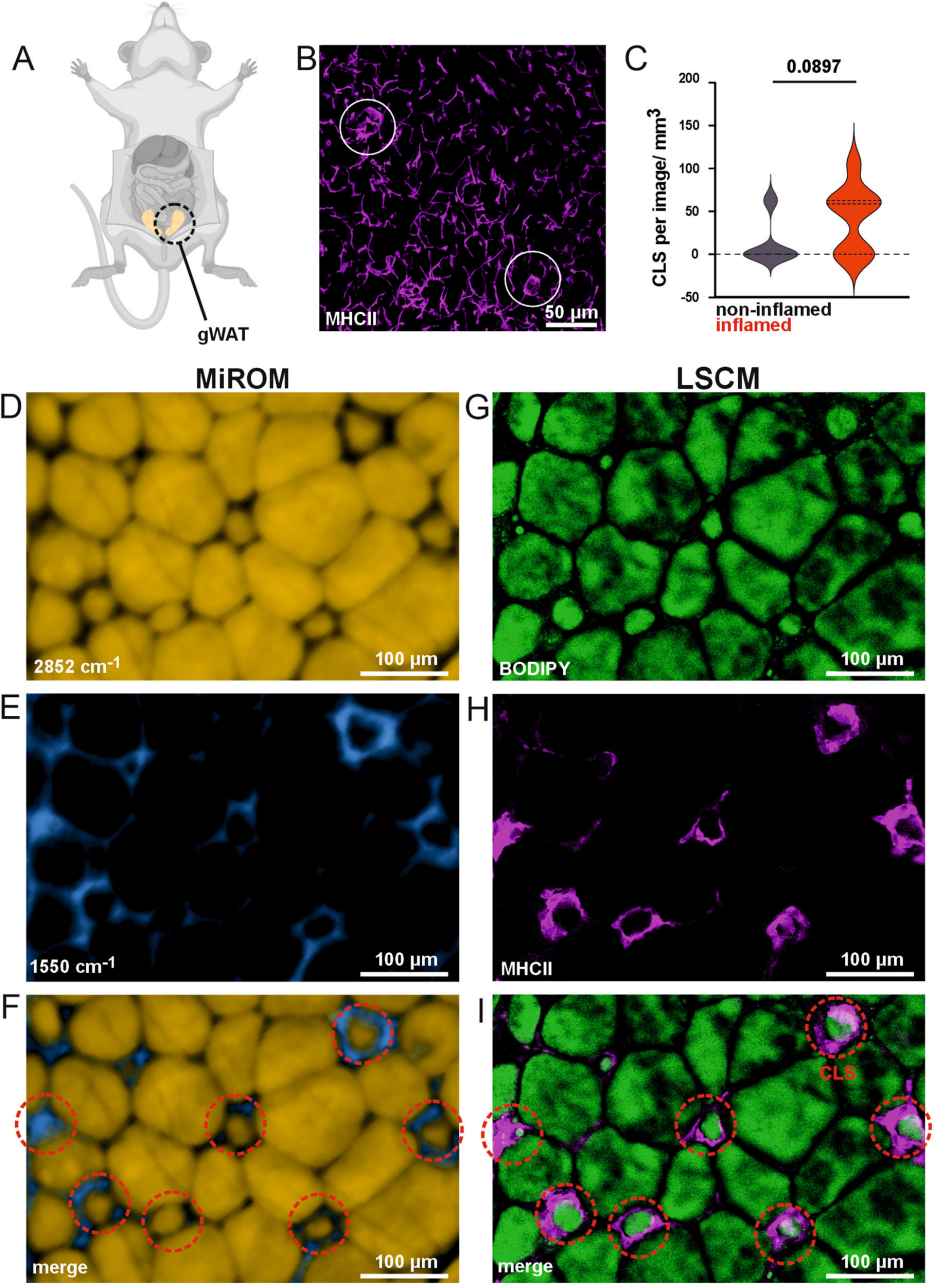
Label-free identification of crown-like structures (CLS) in WAT using MiROM. **A** Illustration of gonadal white adipose tissue (gWAT) extraction area—adipose tissue highlighted in orange. **B** Volumetric image (maximum intensity projection) of gWAT stained for MHC-II (magenta), cleared using our EMOVI approach and z-stacks imaged by LSCM. MHC-II stained macrophages form CLS (white circles) within adipose tissue. **C** Quantitative analysis of CLS in non-inflamed and inflamed adipose tissue. Comparison of MiROM (**D**–**F**) and LSCM (**G**–**I**) imaging modalities for CLS in the same inflamed WAT tissue and FOV. MiROM images are shown with 2852 cm^−1^, 1550 cm^−1^, and combined contrast. FOV: 492 × 328 μm^2^ (cropped from 1 × 2 mm^2^), 5 μm step size. LSCM images show BODIPY staining, MHC-II staining, and merged images. MiROM enables unstained detection of CLS within the given FOV, as corroborated by LSCM with MHC-II/ BODIPY staining. Imaging and tissue processing times in Table 1. Illustration **A** was created with BioRender.com.

Next, in order to validate the usage of MiROM for label-free analytical histology of unprocessed tissues, we determined intrinsic hallmarks of WAT inflammation by MiROM compared to 3D imaging of whole organs using immunofluorescence. To achieve this, we used a mouse model of systemic autoimmunity and immunodeficiency in which mice develop systemic inflammation^26^. Hallmarks of inflammation within WAT include immune cell infiltration, increased expression of pro-inflammatory cytokines and the formation of CLS, which consist of macrophages surrounding dead or dying adipocytes^3^. Typically, the presence of CLS and infiltration of immune cells is quantitatively determined after labeling immune cell subsets with antibodies using histology, immunofluorescence or immunohistochemistry techniques (Supplementary Fig. 6A, B)^4–6^ We applied whole tissue staining and volumetric imaging of whole WAT pieces using EMOVI to determine immune cell infiltration and obtain spatial information on aggregated macrophages forming CLS^27^ as exemplified in Fig. 2A–C. We labeled macrophages in non-inflamed, homeostatic and inflamed WAT isolated from mice using an antibody against the major histocompatibility class (MHC)-II antigens expressed by these immune cells. The cleared and stained WAT was imaged using LSCM. Infiltrated immune cells were quantified using the surface generation tool of IMARIS to analyze the amount of infiltrating labeled cells (Supplementary Fig. 6C, D). CLS are clustered structures clearly recognizable by MHC-II immunolabeling contrast (Fig. 2B). As expected, from visual inspection of equal tissue volumes, we observed an elevated CLS count in inflamed adipose tissue compared to non-inflamed, homeostatic tissue (Fig. 2C).

Unlike immunofluorescence, which requires exogenous labels, MiROM is able to identify CLS in WAT using intrinsic biomolecular contrast only. For instance, Fig. 2D, E shows MiROM micrographs of inflamed WAT at 2852 and 1550 cm^−1^ excitation, revealing the spatial distribution of endogenous lipids and proteins, respectively. For comparison, images of the same field-of-view obtained by LSCM using BODIPY staining (for lipids) and MHC-II antigen labeling (for CLS) are shown in Fig. 2G, H. From Fig. 2D, G (as in Fig. 1D, E), we observed that the MiROM micrograph at 2852 cm^−1^ retrieved the adipocyte distribution in WAT, matching the images obtained by LSCM using BODIPY labeling. Additionally, we observed structures with enhanced protein contrast at 1550 cm^−1^ (Fig. 2E, circa 10% higher CNR compared to the adjacent ECM, see Methods), which spatially overlap with CLSs labeled with antibodies against MHC-II antigens, Fig. 2H. The increased protein contrast might originate from the accumulation of immune cells around the dying adipocyte when a CLS is formed. Figure 2F shows merged images in which several CLS can be identified, as confirmed by LSCM in Fig. 2I (CLS in red-dashed circles). Moreover, as expected, dying adipocytes were observed to be about 50% smaller and generated up to 20% less OA lipid contrast (at 2852 cm^−1^) compared to adjacent (healthy) adipocytes (Fig. 2F). Importantly, for the micrographs shown in Fig. 2, tissue preparation for LSCM required 27 h in total, including BODIPY and anti-MCH-II antigen staining, while MiROM needed only 10 min preparation time (similar to tissue preparation time for micrographs shown in Fig. 1). Here, imaging acquisition for the FOVs shown in Fig. 2D–H, required 12 min (for MiROM) and 10 s (for LSCM) per channel. Thus, despite the fact that MIROM currently requires longer image acquisition time, the overall time needed to identify CLS in WAT using MIROM compared to LSCM is substantially reduced by avoiding the use of external labels; see Table 1 for details.

In addition to the morphological information on CLS, MiROM can also be used to extract spectral information, thus expanding the readouts provided with label-free analytical histology in tissues. For example, Supplementary Figs. 7 and 8 show the spectra of dying adipocytes and immune cell accumulation in the mid-IR excitation range between 2930 and 910 cm^−1^. We compared the spectra with adjacent adipocytes and ECM to see the contribution of specific bonds in CLS. The analysis is congruent with the intensity and map distribution in the micrograph of Fig. 2F. Dying adipocytes displayed a lower intensity in CH_2_ regions compared to adjacent adipocytes (e.g., 1.3-fold lower intensity at 2852 cm^−1^), while immune cell accumulation led to a higher intensity in the protein-related region between 1700–1500 cm^−1^ (≈1.5-fold higher intensity in amide I and II bands compared to ECM), see Supplementary Figs. 7 and 8.

**Fig. 3:**
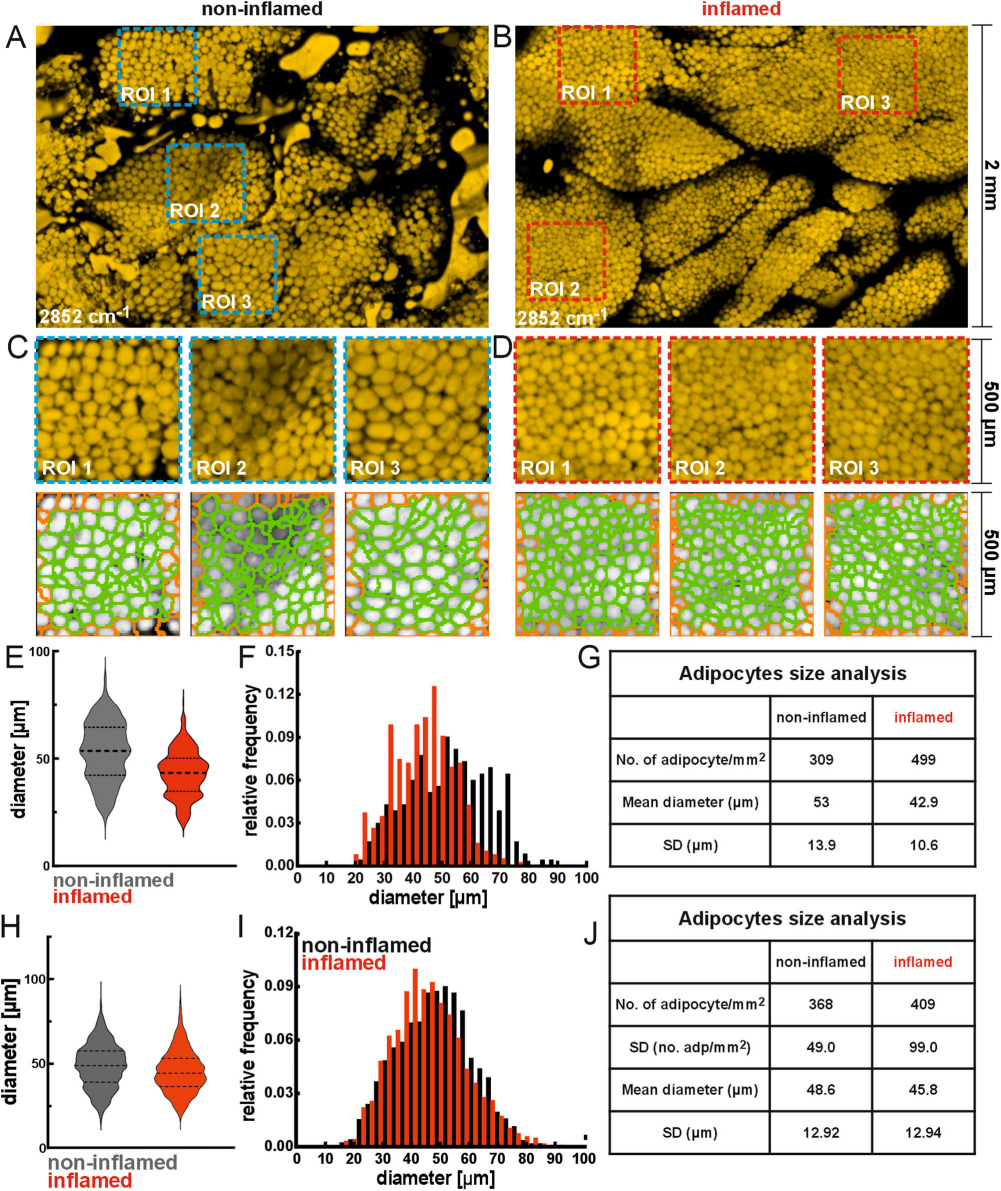
MiROM identifies inflamed WAT by adipocyte size and count. **A**, **B** 2 × 3 mm^2^ micrographs of non-inflamed and inflamed adipose tissue at 2852 cm^−1^, in which the three regions selected for statistical analysis of the cell size distribution are highlighted. **C**, **D** Zoom-in micrographs (FOV: 500 × 500 μm^2^) for the selected regions of interest (ROIs, top row) and corresponding segmentations produced with the CellProfiler 4.0. **E** Violin/box plots of the adipocyte size distribution determined in the two exemplary tissues within the selected ROIs (**C**, **D**). **F** Adipocyte size distribution histograms as relative cell size frequency for the two shown exemplary tissues. Results are summarized in (**G**). **H**–**J** Summary of adipocyte size analysis on 17 mice. Adipocyte size distribution between 8 non-inflamed and 9 inflamed mice showing that the adipocytes in inflamed tissue are on average smaller than adipocytes in non-inflamed tissue. **H** Violin plots with the size distribution, **I** relative size frequency plot, and **J** summary of overall results in 17 mice.

In addition to immune cell infiltration (quantified in Supplementary Fig. 6D, C) and CLS formation, another hallmark of adipose tissue inflammation is changes in adipocyte morphology. Inflammation of WAT, for example, during intestinal inflammation, leads to adipocyte hyperplasia; characterized by reduced adipocyte size and increased adipocyte number^28^. These two morphological features of WAT inflammation can be quickly quantified given MiROM’s high contrast and resolution when imaging adipocytes using excitation at 2852 cm^−1^ without the need of labels. We applied MiROM to quantify the adipocyte size distribution and count in WAT from 17 mice (8 mice with systemic inflammation and 9 control mice). Representative MiROM micrographs (2 × 3 mm^2^ FOV) of WAT samples for the two measured groups are shown in Fig. 3A, B; additionally, zoomed-in micrographs of three selected regions of interest (ROIs) are depicted in Fig. 3C, D. Next, we determined the size and number of adipocytes in the selected ROIs by means of image processing using the CellProfiler 4.0^29^ analysis tool (see Methods) in the 17 samples. In the representative examples (Fig. 3A–D), we measured a larger mean diameter of adipocytes (Fig. 3E) in non-inflamed tissues (53 μm in control mice) compared to inflamed tissues (43 μm in mice with systemic inflammation). Additionally, we found an increased adipocyte count (number) per mm^2^ in inflamed tissue compared to non-inflamed WAT (499 versus 309 adipocytes/mm^2^, respectively). Interestingly, by illustrating the relative frequency of adipocyte size in the measured tissues, for the two representative samples, we detected a larger population of adipocytes with large diameters ranging from 60 to 80 μm in non-inflamed tissue compared to inflamed tissue, Fig. 3F, G summarizes the adipocyte size distribution and adipocyte count in inflamed and non-inflamed WAT obtained with MiROM for the examples in Fig. 3A–D. Results for measurements from a sample set of 17 mice are shown in Fig. 3H–J. Here (similar to Fig. 2E–G), we observed a larger adipocyte count in inflamed tissue than in non-inflamed WAT—409 versus 368 adipocytes/mm^2^, respectively. Moreover, the size distribution of non-inflamed adipocytes was found to cover larger diameter values than inflamed adipocytes, diameters with highest frequencies between 45 and 60 μm compared to 35 and 50 μm, respectively. As summarized in Fig. 3J, the mean adipocyte diameter of the complete set of tissues was found to be 48.6 μm for non-inflamed tissue and 45.8 μm for inflamed tissue—reflecting a considerable heterogeneity of adipocyte sizes in WAT.

**Fig. 4:**
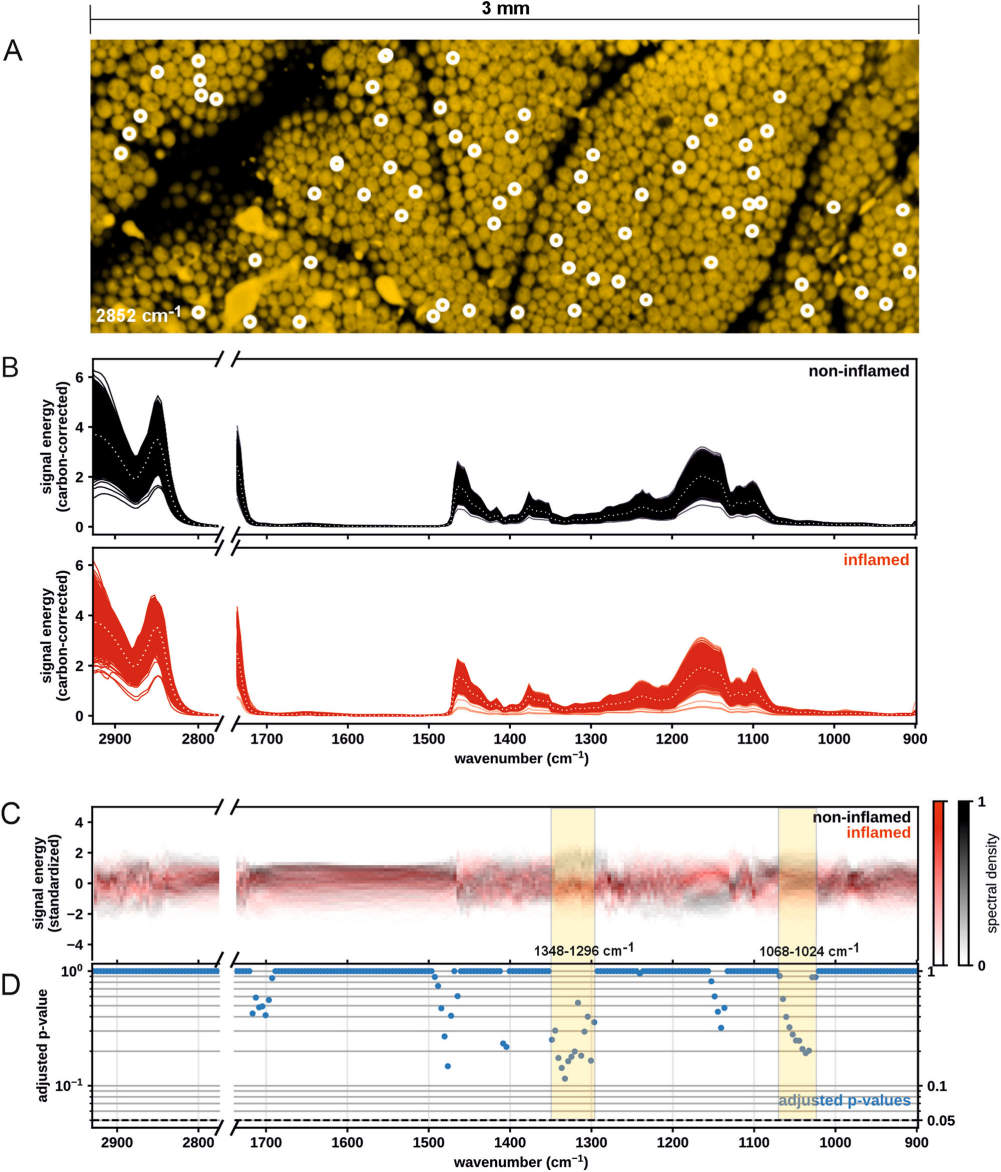
Exploring spectral differences between inflamed and non-inflamed WAT with MiROM. **A** 1 × 3 mm^2^ micrograph at 2852 cm^−1^ of WAT with randomly selected adipocytes marked by white circles. **B** Spectra of adipocytes in non-inflamed and inflamed tissues. The mean spectra of the groups are shown with dotted lines. **C** Spectral density plot (two-dimensional histogram) of standardized adipocyte spectra with color coding for tissue type. Non-inflamed tissue spectral density is shown on a white-black scale, while the inflamed tissue spectral density is shown on a white-red scale. **D** *p*-values of Welch *t*-tests between inflamed and non-inflamed tissues for each recorded wavenumber (Bonferroni corrected). The dashed line shows the statistical significance threshold of 0.05.

Finally, taking advantage of the imaging and spectroscopy abilities of MiROM, we explored the mid-IR spectral differences between inflamed and non-inflamed WAT. For this purpose, we acquired MiROM spectra of randomly selected adipocytes in WAT samples from 17 mice (9 inflamed and 8 non-inflamed as control; as mentioned above), collecting 2182 artifact-free adipocyte spectra in total; see Methods section for details. Figure 4A shows a representative example of a MiROM micrograph of WAT with selected adipocytes (indicated by white circles). Figure 4B shows the resulting spectra from 1083 inflamed and 1099 non-inflamed WAT adipocytes.

The recorded spectra were pre-processed by calculating OA signal energies and then applying L^1^-normalization and spectral standardization to the resulting spectra while removing outliers, as discussed in the Methods section. L^1^-normalization scales the spectra by the sum of all entries (signal energies) in the spectrum, so that all spectra have an identical value for total energy after the operation. Normalization thereby compensates for day-to-day laser power and OA conversion efficiency fluctuations. After normalization, only relative spectral variations between wavenumbers remain. Additionally, spectral standardization scales the data at every wavenumber to a similar range of values to remove any bias in the signal intensity. Standardization is relevant, as a strong signal at a specific wavenumber does not necessarily imply importance of that wavenumber for distinguishing inflamed cells from non-inflamed cells.

Figure 4C depicts a 2D plot of adipocyte spectral density against standardized OA signal energy (y-axis). We independently color-coded the spectra of inflamed and non-inflamed adipocytes red and black, respectively. Wavenumber ranges with reduced spectral overlap between mice with or without inflammation can be observed; for example: 1350–1290 cm^−1^ and 1060–1040 cm^−1^, indicated in Fig. 4C, D. However, for most wavelengths, the spectral distributions of the two types mostly overlap.

Exploratory data analysis based on the Uniform Manifold Approximation and Projection (UMAP)^30^ algorithm showed a strong clustering of the spectra belonging to each mouse, indicating a substantial statistical interdependence of the cell spectra acquired for each mouse (see Supplementary Fig. 9). All subsequent statistical analyses were therefore performed on averaged spectra from each mouse.

To statistically identify spectral differences between the mice with or without inflammation, we performed a wavenumber-wise Welch t-Test on the empirical means of the spectra obtained from inflamed and non-inflamed tissue. To compensate for multiple comparisons, we estimated the number of effective degrees of freedom in the averaged spectra by fitting a Principal Component Analysis (PCA) and adding components until explained variance reached 99%. We took the number of needed PCA components (n_c_ = 12) as the effective number of degrees of freedom of the data and performed a Bonferroni correction of obtained *p*-values. Figure 4D shows the adjusted p-values of the Welch *t*-Tests for all recorded wavenumbers. Although no significant statistical differences between inflamed and non-inflamed groups were found (*p* > 0.05 for all wavenumbers), several wavenumbers exhibit *p*-values below 0.2, suggesting that significant spectral differences might be verifiable with a larger sample size—see Table 2.

**Table 2:** Wavenumbers with low adjusted *p*-values when inflamed and non-inflamed WAT were compared.

Wavenumber (cm^−1^)	1476.55	1340.55	1336.55	1332.55	1328.55	1324.55	1320.55	1312.55	1300.55	1036.55
Adjusted *p*-value	0.148	0.175	0.143	0.115	0.164	0.179	0.199	0.183	0.166	0.193

## Discussion

We applied MiROM for the label-free, non-destructive detection of morphological hallmarks of WAT inflammation, namely CLS and adipocyte hyperplasia, using mid-IR spectral features as intrinsic contrast sources instead of immunohistochemistry or immunofluorescence labels. Additionally, we explored the spectral differences between adipocytes in inflamed and non-inflamed WAT. CLS develop in inflamed fat tissue when dying adipocytes are enclosed by infiltrating macrophages, which can be detected by MiROM using intrinsic lipid (at 2852 cm^−1^, CH_2_ band) and protein (at 1550 cm^−1^, amide II band) vibrational contrast. Imaging at the amide II absorption band of proteins, we detected accumulation of macrophages as increased contrast around dying adipocytes, as confirmed by LSCM using MHC-II antigen labeling. In lipid maps of inflamed WAT, we observed a reduction in the size of adipocytes surrounded by macrophages compared to adjacent (healthy) adipocytes. Additionally, we noticed reduced lipid contrast indicating a diminished lipid concentration in dying adipocytes compared to healthy adipocytes. Similar to findings using histology^28^, we found a reduced mean diameter of adipocytes in inflamed tissue compared to non-inflamed adipocytes using MiROM. More importantly, MiROM notably reduced the overall time needed to identify hallmarks of WAT inflammation up to 24-fold by simplifying the tissue processing pipeline—as summarized in Table 1.

Nevertheless, although clustering of macrophages was detected by MiROM with approximately 10–20% higher CNR at amide I and II bands compared to the CNR of ECM, some CLS were difficult to localize without the aid of reference structures, such as dying adipocytes. Additionally, the CNR threshold to define clustering immune cells might differ from sample to sample, increasing the risk of misidentification of CLS. To avoid this risk, the specificity of MiROM could be further enhanced, for example, by acquiring hyperspectral images in combination with mass spectrometry, which could serve as a reference to fully disclose the biomolecular composition of the inflamed WAT. This would allow us to establish a correlation between the biomolecular composition of inflamed WAT and its hyperspectral content (i.e., with specific spectral chemical bond features) and to define the basis for developing a spectral-feature-based model for fast identification of inflamed WAT. Additionally, expanding the study to a larger sample size than the one reported here (*n* = 17) might contribute to the identification of more specific and significant spectral differences between inflamed and non-inflamed WAT—enabling the generation of classification models from hyperspectral readouts. It should be noted that excluding sample preparation times, system preparation and imaging times of MiROM is overall more time consuming and slower than those of commercial LSCM systems (see Table 1). Thus, great efforts are presently focused on developing faster system preparation protocols and imaging acquisition for MiROM. For example, in order to boost imaging speed, we are implementing onboard averaging of OA signals to reduce data-transfer time, improving our scanning imaging-reconstruction algorithm to take full advantage of the fast laser repetition rate of our QCL (100 Khz), and implementing in-parallel acquisition of multiple molecular contrast by simultaneous QCL excitation at different wavenumbers. Other strategies we are exploring include: upgrading our QCL to faster laser repetition rates (up to 2 Mhz commercially available) in combination with galvo-mirror-stage scanning, and sparse raster-scanning in combination with Bayesian imaging reconstruction.

The presence of CLS in WAT of the breast has been associated with a higher risk of breast cancer^31^ and a reduced time to metastatic disease in treated breast cancer patients with high body mass index^32^. A fast, label-free, non-destructive assessment of the inflammatory state and tissue morphology of the unprocessed WAT using MiROM might, in future stages of the technology, be applied to decide on the area of resection of the tumor border (e.g., of breast cancer but potentially also pancreatic cancer), or inflamed WAT during Crohn’s disease. Furthermore, determining CLS with 3D methods such as MiROM or whole organ imaging might be a prognostic tool to determine response to therapy (e.g., during breast cancer treatment). Overall, this study sets the foundation for the application of MiROM as an enabling analytical tool with chemical bond-selective contrast for fast, label-free, non-destructive assessment of the inflammatory state of unprocessed WAT.

## Methods

### MiROM experimental array and tissue measurements

MiROM works in transmission mode. The excitation beam was confocally aligned with the detector as shown in Fig. 1A. A QCL (MIRcat, Daylight Solutions) was the source of MIR light, which was focused on the sample using a 0.5 NA 36X objective (Newport Corporation). The QCL covered the spectral range from 3.4–11 µm (2932–909 cm^−1^) with a spectral linewidth full width at half maximum (FWHM) of <1 cm^−1^. The focused mid-IR light went through the bottom part of a custom-made Petri dish, which is made out of a ZnS window (Crystal GmbH) for mid-IR transmission. The sample examined was irradiated with the focused mid-IR beam with a pulse width of 20 ns and, following absorption, generated an optoacoustic signal, which traveled through the acoustic coupling and was detected by the focused ultrasound transducer (Sonaxis) with a 25 MHz central frequency and with a focal length of 6 mm. The dish holder was mounted on motorized stages (Physik Instrumente), allowing movement in the x-y direction, while the transducer and objective were each attached to a z-direction mechanical stage to allow for optical and acoustic focusing, respectively.

The lateral optical resolution of our microscopy system was experimentally measured by imaging synthetic polyethylene microspheres (1 µm diameter) embedded in agar in order to obtain the point-spread-function used to determine the resolution. Our experimental resolution assessment revealed that at 2850 cm^−1^ (3508 nm)—relevant for lipid detection—the lateral resolution is 5.3 μm. Technical details on the optical performance of MiROM were reported previously elsewhere^18^.

Before tissue is placed in the modified dish for measurement, the buffer solution from the surface of tissue was removed for minimizing the water absorption. The tissue is then placed on top of the ZnS window of the modified Petri dish and covered with an acoustically transparent membrane. The tissue was held tightly against the ZnS window by a custom-made Polyvinyl Chloride (PVC) tissue holder. Figure 1A shows the final assembly of the dish, with the sample confocally aligned with the light source and ultrasound detector, allowing OA signal detection in the focal spot.

### MiROM image processing

Tissue imaging was performed by raster-scanning a FOV (i.e., 2 × 3 mm^2^ or 2 × 2 mm^2^) with a 5 µm step size and OA signals were acquired at each position. OA signals were acquired for wavenumbers from the 2932–2772 cm^−1^ and the 1736–910 cm^−1^ ranges. For each scanning position *x* and wavenumber *λ*, fifty OA signals were acquired and averaged to form the optoacoustic signal ${{OAS}}_{x,\lambda }\left(t\right)$, at an excitation laser pulse frequency of 100 kHz—resulting in an acquisition time of 500 μs per pixel. Each pixel on MiROM micrographs is then assigned the peak-to-peak amplitude ${ptpOA}{S}_{x,\lambda }$ of the ${{OAS}}_{x,\lambda }\left(t\right)$ recorded at the pixel location *x*. For this configuration, for example, the image in Fig. 1F–H acquired for a 2 × 3 mm^2^ FOV and 5 µm step size (later cropped to 2 × 2 mm^2^ for a zoomed-in view), was taken in approximately 35 min per excitation wavenumber. Likewise, the micrographs in Fig. 2D, E, taken from a 1 × 2 mm^2^ FOV at 5 µm step size and cropped to 328 × 492 mm^2^ for zoom-in views, were taken in circa 12 min per wavenumber. The micrographs were subsequently processed with ImageJ (ImageJ 2.9.0). The raw image was used to calculate Contrast-Noise-Ratio values (${\rm{CNR}}=\frac{{\rm{ptpOA}}{{\rm{S}}}_{{\rm{A}}}-{\rm{ptpOA}}{{\rm{S}}}_{{\rm{B}}}}{{\rm{Nois}}{{\rm{e}}}_{{\rm{ptp}}}}$) where ptpOAS_A_ is the (peak-to-peak) optoacoustic contrast in a point (A) on the sample, ptpOAS_B_ is the optoacoustic contrast in a point (B) in the background, and Noise_ptp_ is the dark noise of the system (approximately 1.3 mV, defined as the average intensity of the OA signal used for imaging when the excitation beam is blocked). For example, Fig. 1F shows a maximum CNR in the lipid channel of $\frac{460{\rm{mV}}-12{\rm{mV}}}{1.3{\rm{mV}}}=\frac{344}{1}$. While in the protein channel, CLS showed approximately 10% higher CNR compared to ECM; namely, 54:1 and 48:1, respectively.

Unless otherwise stated, and in order to enhance contrast and structural definition, all MiROM micrographs in the figures displayed in the manuscript are the normalized square intensity of the OA signal, namely ${\rm{NOA}}{{\rm{S}}}_{{\rm{pixelx}},{\rm{\lambda }}}^{2}=\frac{{{\rm{ptpOAS}}}_{{\rm{pixelx}},{\rm{\lambda }}}^{2}}{\mathop{\max }\nolimits_{{\rm{x}}^{\prime}}\left({\rm{ptpOA}}{{\rm{S}}}_{{\rm{FOVx}}^{\prime},{\rm{\lambda }}}^{2}\right)}$. A comparison between linear and square normalized contrast MiROM micrographs is now shown in Supplementary Fig. 10. To determine the adipocyte size distributions, the NOAS^2^ micrographs acquired at 2852 cm^−1^ were analyzed with the CellProfiler 4.0 pipeline “CP_adipocyteanalysis.cpproj”. Here, the adipocytes were assumed to span between 3 and 20 pixels diagonally, corresponding to diameters between 15 and 100 µm, respectively. Adipocyte boundaries were extracted based on the NOAS² intensity distribution and corresponding adipocyte diameters were calculated within the CellProfiler software.

### Acquisition of MiROM spectra

MiROM spectra were obtained by recording OA signals for multiple wavenumbers in selected positions of a given FOV. For the spectra presented in this work, OA signals were recorded at wavenumber ranges 2932–2772 cm^−1^ and 1736–910 cm^−1^ at step sizes of 4 cm^−1^. To increase the SNR, the OA signals acquired for use in the spectral analysis were averaged over 10000 laser pulses per wavenumber at each selected adipocyte.

From these OA signals, two types of spectra were calculated: (1) peak-to-peak signal amplitude spectra ${\left({Spe}{c}_{x}^{{ptp}}\right)}_{\lambda }={ptpOA}{S}_{x,\lambda }$, and (2) signal energy spectra ${Spe}{c}^{{SE}}$. For the latter, we first removed any DC offset from the OA signals ${{OAS}}_{x,\lambda }\left(t\right)$. The DC offset ${S}_{x,\lambda }^{{DC}}$ was estimated from the initial 176 data points of the signal transient (A-line), where no OA signal is generated. The signal energy spectra were then calculated as ${\left({Spe}{c}_{x}^{{SE}}\right)}_{\lambda }={\sum }_{i=176}^{400}{\left[{OA}{S}_{x,\lambda }\left({t}_{i}\right)-{S}_{x,\lambda }^{{DC}}\right]}^{2}$. Each adipocyte spectrum was acquired in approx. 4 min. To compensate for the emission intensity profile of the QCL, we corrected samples’ OA spectra by dividing each spectra by the OA spectrum measured for carbon tape. Carbon tape was used in this case because it has a relatively broadband absorption spectrum in the emission range of the QCL, therefore allowing a direct measurement of the spectral intensity profile of the laser.

### Search for spectral differences between inflamed and non-inflamed WAT adipocytes

In order to explore spectral differences between adipocytes in inflamed and non-inflamed WAT, we recorded MiROM spectra of randomly selected adipocytes for each of the WAT samples examined (9 inflamed and 8 non-inflamed as control, as mentioned above). For each adipocyte *a* measured, we acquired spectral data and computed the signal energy spectrum ${Spe}{c}_{{x}_{a}}^{{SE}}$.

To prepare measured spectra for analysis, we additionally performed the following pre-processing steps:

1. The QCL laser exhibited consistent wavenumber tuning errors in the 2932–2772 cm^−1^ wavenumber range for some tissues, resulting in a shift of the acquired spectra in the wavenumber dimension. As the shift was found to be identical for all affected tissues, it was corrected by linearly interpolating the acquired spectra at the nominally targeted wavenumbers.
2. As the signal energy spectra were affected by day-to-day fluctuation in the end-to-end energy transfer efficiency of the system (laser power fluctuations, slight variations in the co-alignment quality of the optic and acoustic focus, and Grüneisen parameter changes with temperature dependency), the absolute scale of their signal energies was not consistent. However, the relative spectral intensities, given as the signal energy ratios of pairs of wavenumbers, were not affected by this fluctuation, given that the energy transfer efficiency of the system stayed constant within the time required to acquire a single spectrum. We, therefore, focused on studying changes in the relative spectral intensity caused by inflammation. To highlight these changes, we normalized all signal energy spectra to a constant total spectral energy of one by dividing the spectra by their sum over all wavelengths: ${Spe}{c}_{{x}_{a}}^{{SE},{norm}}=\frac{{Spe}{c}_{{x}_{a}}^{{SE}}}{{\sum }_{\lambda {\prime} }{Spe}{c}_{{x}_{a},\lambda {\prime} }^{{SE}}}$.
3. Some spectra contained artifacts or faulty acquisitions, necessitating the removal of outliers. For this, we employed a combination of outlier detection routines: *LocalOutlierFactor*^33^ from the *scikit-learn* Python package and wavenumber-wise thresholding. The outlier detection was tuned to select obvious outliers while retaining most spectra recorded (89.2%).
4. Since many analysis methods expect standard Gaussian distributed input data, we applied a standardization routine from the *scikit-learn*^34^ package to the corrected and normalized signal energy spectra. This offset and scaled the spectral values for each wavenumber independently, so that they had a mean of zero and a unit standard deviation in each wavenumber. This method highlighted relative differences in the spectral intensity among all recorded adipocyte spectra. Finally, we weighted the spectra by the inverse count of spectra from their respective tissue in the standardization procedure to prevent biasing the standardization when more spectra were recorded for certain tissues.

The resulting corrected, normalized and standardized spectra ${Spe}{c}_{{x}_{a}}^{{SE},{st}}$ were the basis of all subsequent analyses. Figure 4C shows the empirical distribution of the standardized spectrum values for inflamed and non-inflamed tissues as a function of the wavenumber *λ*.

To study which factors drive similarity and dissimilarity in the recorded adipocyte spectra, we employed a UMAP projection of the standardized spectra into a two-dimensional space. We separately color-coded the embedded points (corresponding to an adipocyte spectrum each) by the inflammation status of the donated tissue, the acquisition time, and the tissue ID. This revealed a strong clustering of the spectra by tissue sample, indicating that the spectra recorded on a single tissue were not statistically independent, but rather contain tissue-specific features that separate them from the adipocyte spectra of other tissue samples. Correspondingly, we cannot treat the adipocyte spectra as statistically independent observations in the comparison between inflamed and non-inflamed tissue.

To create representative spectra for each tissue, we averaged the adipocyte spectra belonging to each tissue and continued the analysis on the per-subject averaged tissue spectra (psaTS). Statistical testing for differences in the distribution mean of inflamed and non-inflamed tissue adipocyte spectra was performed using the Welch *t*-test. For each wavenumber recorded, an independent test for differences in the population means of the tissue groups was performed. These tests yielded raw *p*-values for each wavenumber.

Given the large number of parallel statistical tests, correction for multiple testing was necessary. The t-tests treat the value distributions at each wavenumber as statistically independent, suggesting a Bonferroni correction of the *p*-values. However, the observed spectra follow smooth curves with only a few jumps, implying strong correlations between neighboring wavelengths. Consequently, the spectra had fewer independent degrees of freedom than wavelengths recorded, and a naïve Bonferroni correction with ${n}_{d.o.f.}={n}_{{wavenumbers}}$ would imply an undue burden of proof.

To estimate the effective number of degrees of freedom of the averaged adipocyte spectra dataset, we employed a PCA embedding of the dataset and added PCA components until 99% of the dataset variance was explained. The corresponding number of PCA components was taken as the effective number of degrees of freedom of the averaged adipocyte spectra dataset and used in the Bonferroni correction of the raw *p*-values, yielding the *p*-values presented in Fig. 4C.

### Mouse tissue preparation

*Wipf1*^*−/−*^ mice^35^ were provided by Dr. Raif Geha (Boston’s Children Hospital, Boston, USA) and these mice had been backcrossed on C57BL/6 mice for at least 8 generations. C57BL/6 WT mice were bought from Charles River and bred at the Specific Opportunist Pathogen Free (SOPF) animal facility at the TranslaTUM. Mice aged 16–19 weeks were used for all experiments. All mouse experiments were performed in accordance with the guidelines of the Federation of European Laboratory Animal Science Association (FELASA) and followed the legal approval of the Government of Upper Bavaria (Regierung von Oberbayern).

### Sample preparation

Epididymal WAT was harvested and placed in PBS for immediate use as freshly excised tissue. Tissues were harvested from 17 mice. After harvesting each tissue, the tissue was immediately fixed with IC fixation buffer (Thermo Fisher FB001) for 1 h at room temperature and subsequently stored in 25% (v/v) fixation buffer in PBS at 4 °C until MiROM measurements.

### Immunofluorescence, Histology

After MiROM measurements, the WAT was blocked in Blocking Buffer (1% (v/v) FCS, 1% (v/v) mouse serum and 0.3% (v/v) Triton X-100 in PBS) for 1 h followed by staining using BODIPY™ FLC16 (1:200, Thermo Fisher D3821) for 1 h at 37 °C. The tissue was washed in PBS and imaged in 8 well µ-slides (ibidi, 80826).

For visualization of CLS, the WAT was blocked in Blocking Buffer (1% (v/v) FCS, 1% (v/v) mouse serum and 0.3% (v/v) Triton X-100 in PBS) for 1 h and stained with BV421 anti-MHC II antigen (1:200, Biolegend 107632), SytoxTM Orange (1:50000, Thermo Fisher S34861) and BODIPY™ FLC16 (1:200, Thermo Fisher D3821) for 24 h at 37 °C. The stained tissue was then washed for 1 h at 37 °C in PBS and imaged in 8 well µ-slides (ibidi, 80826).

Whole-mount tissue imaging of WAT was performed to analyse the CLS count and monocyte/macrophage infiltration. Harvested tissue was prepared and cleared as previously described^13^. In short, after fixation WAT was blocked; stained with AF488 anti-MHC II antigen (1:200, Biolegend 107615), BV421 anti-CD163 (1:200, Biolegend 155309), AF647 anti-CD206 (1:200, Biolegend 141711) and AF594 anti-CD11b (1:200, Biolegend 101254) for 72 h at 37 °C; washed; dehydrated using increasing dilutions of isopropanol (Sigma-Aldrich) and cleared using undiluted ethyl cinnamate (Sigma-Aldrich #W243000).

For histology, WAT was fixed in 4% paraformaldehyde for 24 h, dehydrated and embedded in paraffin (Leica ASP 300 S). Blocks were cut into 3.5 µm thick sections, deparaffinized, rehydrated through a graded ethanol series and stained with hematoxylin and eosin (H&E). Subsequently, the H&E-stained slides were dehydrated, cleared in xylene and mounted with coverslips before being scanned using a slide scanner (AT-2, Leica Biosystems) and representative images were collected using Aperio Imagescope software (version 12.3, Leica Biosystems).

### Confocal imaging and image analysis

Imaging was performed by an inverted Leica TCS SP8 confocal microscope with white light laser and HyD photodetectors using an HC PL APO CS2 20X/0.75 IMM objective (Leica Microsystems). The analysis of confocal images and contrast adjustment was performed using Imaris (Bitplane) version 9.5. The Surface Creation Wizard in Imaris was used for binary masking of macrophages. Statistics of objects were exported for cell quantification.

## Supplementary information

Supplementary information

## Data Availability

The datasets used and/or analysed during the current study available from the corresponding author on reasonable request.
