# Author Correction: Fast histological assessment of adipose tissue inflammation by label-free mid-infrared optoacoustic microscopy

**DOI:** 10.1038/s44303-025-00103-0

**Published:** 2025-08-13

**Authors:** Vito Ko, Marie C. Goess, Lukas Scheel-Platz, Tao Yuan, Andriy Chmyrov, Dominik Jüstel, Jürgen Ruland, Vasilis Ntziachristos, Selina J. Keppler, Miguel A. Pleitez

**Affiliations:** 1 Chair of Biological Imaging at the Central Institute for Translational Cancer Research (TranslaTUM), School of Medicine, Technical University of Munich, Munich, Germany; 2 Institute of Biological and Medical Imaging, Helmholtz Zentrum München, Neuherberg, Germany; 3 Institute for Clinical Chemistry and Pathobiochemistry, Technical University of Munich, School of Medicine, Munich, Germany; 4 TranslaTUM, Center for Translational Cancer Research, Technical University of Munich, Munich, Germany; 5 Institute of Computational Biology, Helmholtz Zentrum München, Neuherberg, Germany; 6 German Cancer Consortium (DKTK), Heidelberg, Germany; 7 German Center for Infection Research (DZIF), Munich, Germany; 8 Munich Institute of Biomedical Engineering (MIBE), Technical University of Munich, Garching b. München, Germany; 9 Division of Rheumatology and Clinical Immunology, Medical University Graz, Graz, Austria

**Keywords:** Biochemistry, Biotechnology, Chemistry, Analytical chemistry, Biochemistry, Applied optics, Optics and photonics, Optical techniques, Imaging and sensing, Microscopy, Optical spectroscopy, Immunology, Engineering, Biomedical engineering

Correction to: *npj Imaging* 10.1038/s44303-023-00003-1, published online 06 December 2023

‘In this article the ‘Competing interests statement’ statement was incorrectly given as “V.N. and M.A.P. are founders and equity owners of sThesis GmbH. V.N. is a founder and equity owner of iThera Medical GmbH, of Spear UG and of I3 Inc”

but should have been “V.N. and M.A.P. are founders and equity owners of sThesis GmbH. V.N. is a founder and equity owner of iThera Medical GmbH, of Spear UG and of I3 Inc. The remaining authors declare no competing interests”’. The original article has been corrected.

