# Supplementary material for: Fast histological assessment of adipose tissue inflammation by label-free mid-infrared optoacoustic microscopy
Source: Npj Imaging. 2023 Dec 6;1:3. doi: 10.1038/s44303-023-00003-1 (PMC11041735; doi:10.1038/s44303-023-00003-1)
Supplement: Supplementary file 1 — Supplementary information [file 44303_2023_3_MOESM1_ESM.pdf]

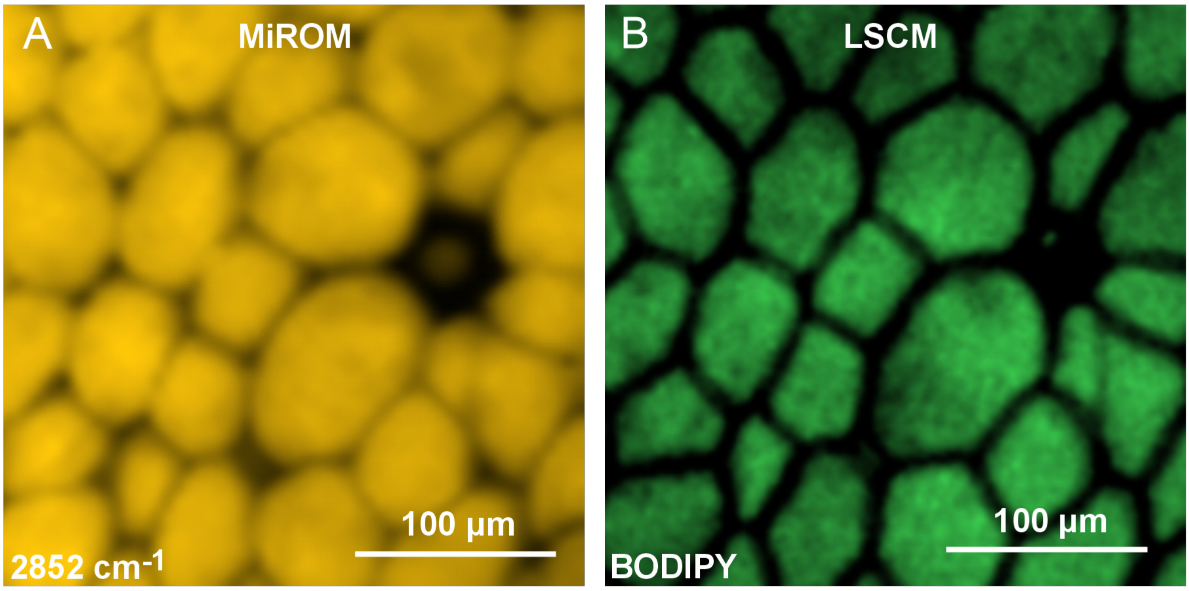

Supplementary Fig. 1: Comparison between MiROM and LSCM. (**A, B**) Zoom-in from **Fig. 1D** and **E**, showing high structural agreement between MiROM and LSCM with BODIPY labelling.

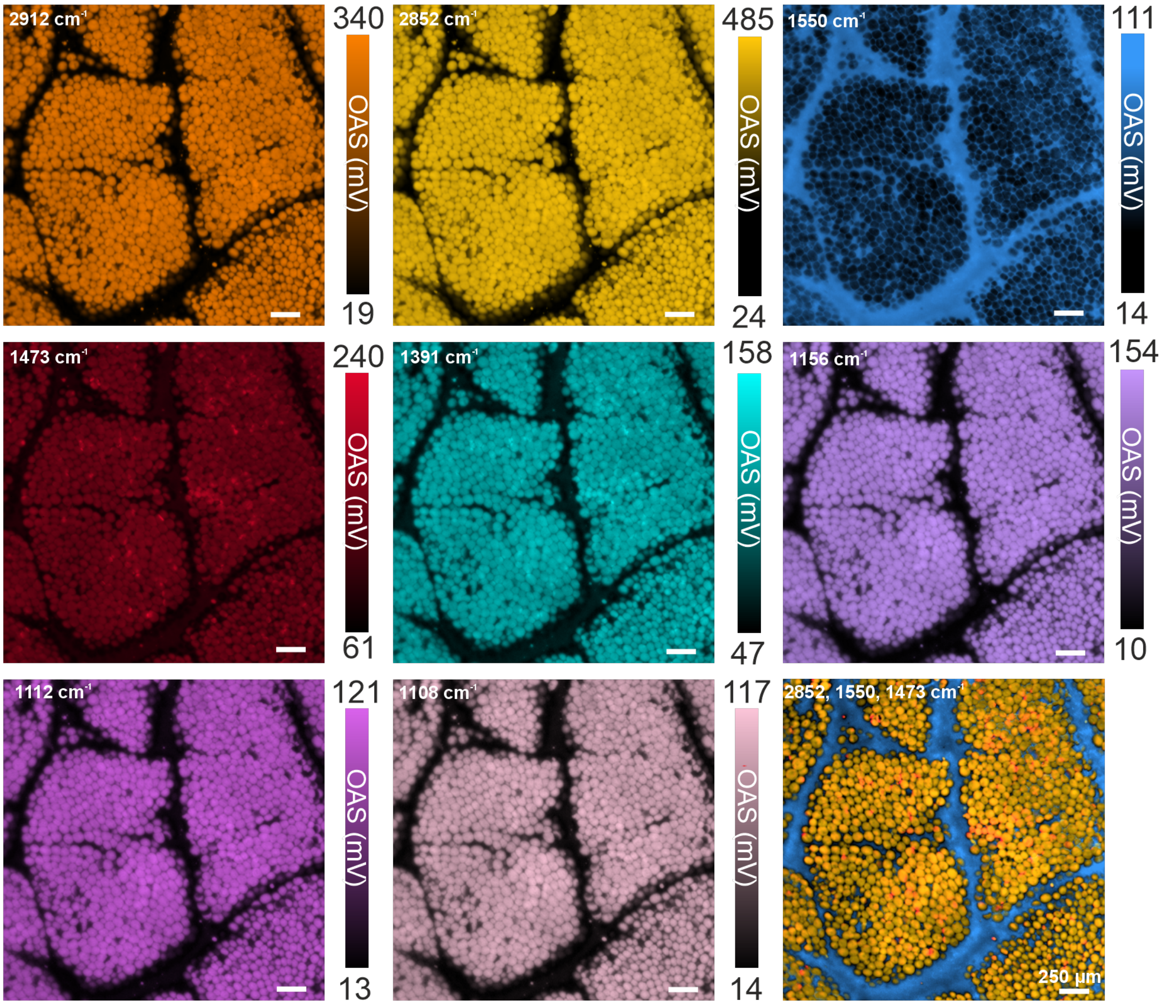

Supplementary Fig. 2: WAT imaging at multiple wavenumbers. Unprocessed micrographs from MiROM at 8 wavenumbers with peak-to-peak intensity contrast bar of optoacoustic signal in mV.

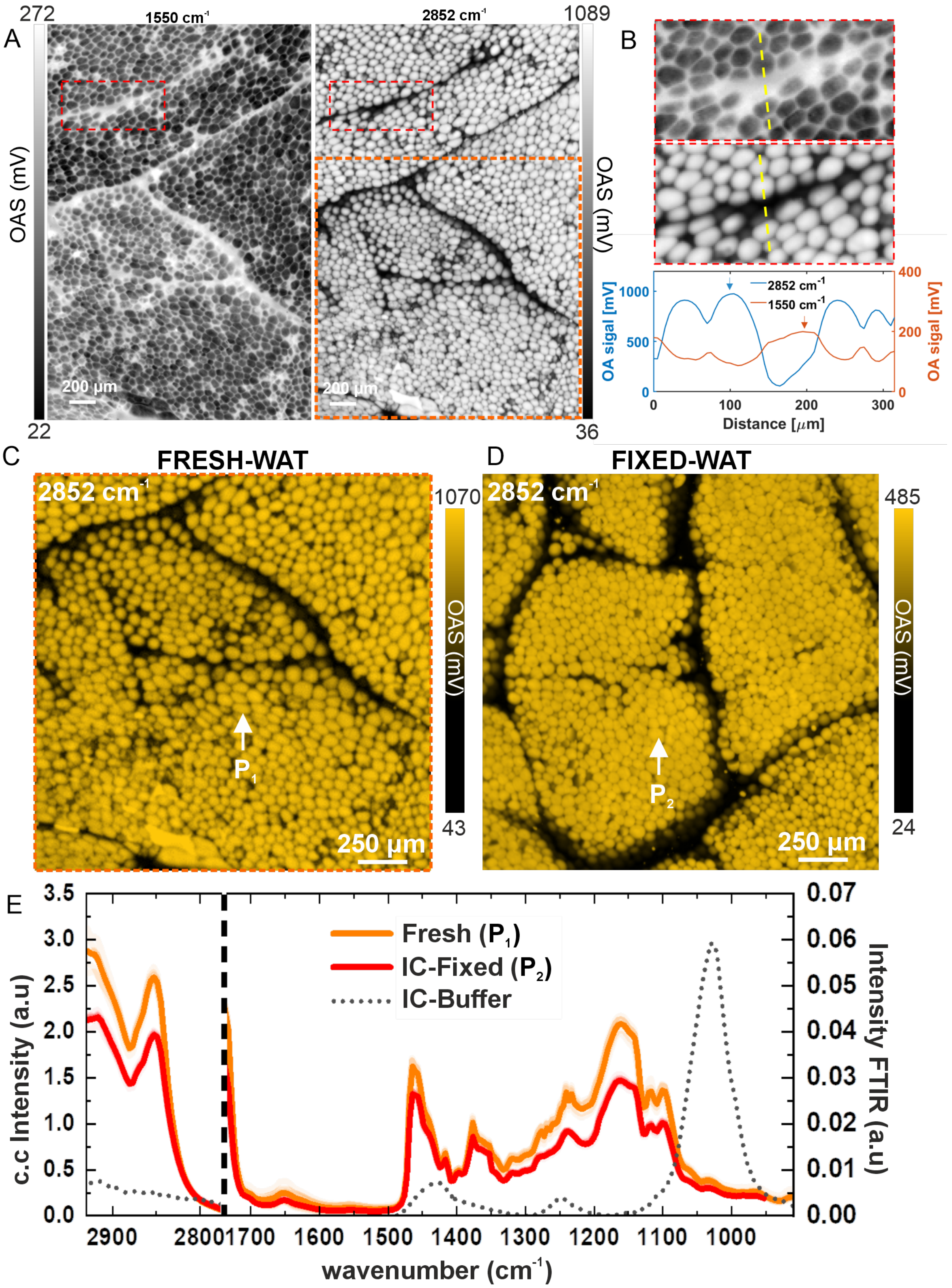

Supplementary Fig. 3: Comparison of MiROM contrast in fresh and fixed WAT. (**A**) Imaging of fresh-WAT at two different wavenumber 1550 and 2852 cm^{-1}. (**B**) The contrast of the tissue has inverse correlation between 1550 and 2852 cm^{-1}, showing the hydrophobic characteristic of lipid droplet. (**C-E**) The fixation's effect on the tissue in terms of image contrast and spectra of adipocytes. (**C**) Fresh and (**D**) fixed white adipose tissue—wild type (non-inflamed), show similar morphology in adipocyte contrast of 2852 cm^{-1}. (**E**) MiROM spectra from fresh and fixed WAT compared to IC-buffer spectra—no spectral features from IC-buffer (for example, 1020 cm^{-1}) was observed in fixed tissues.

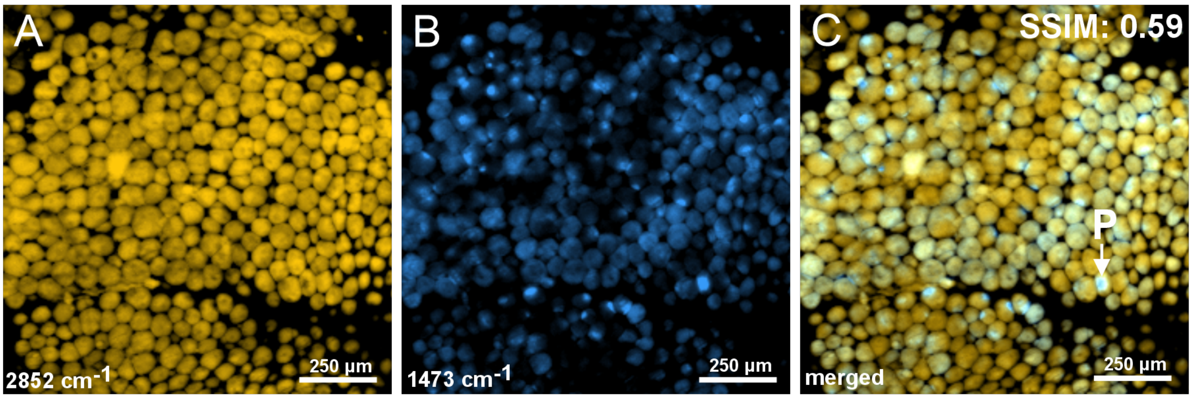

Supplementary Fig. 4: Comparison between 2852 cm^{-1} and 1473 cm^{-1} micrographs. (A, B) Individual contrast from merged micrograph of **Fig 1I**, showing a highlight of small red features from 1473 cm^{-1} channel. Structural similarity index measure (SSIM) of both channels shows the value of 0.59. **(C)** merged of both channels highlighting the features at 1473 cm^{-1}.

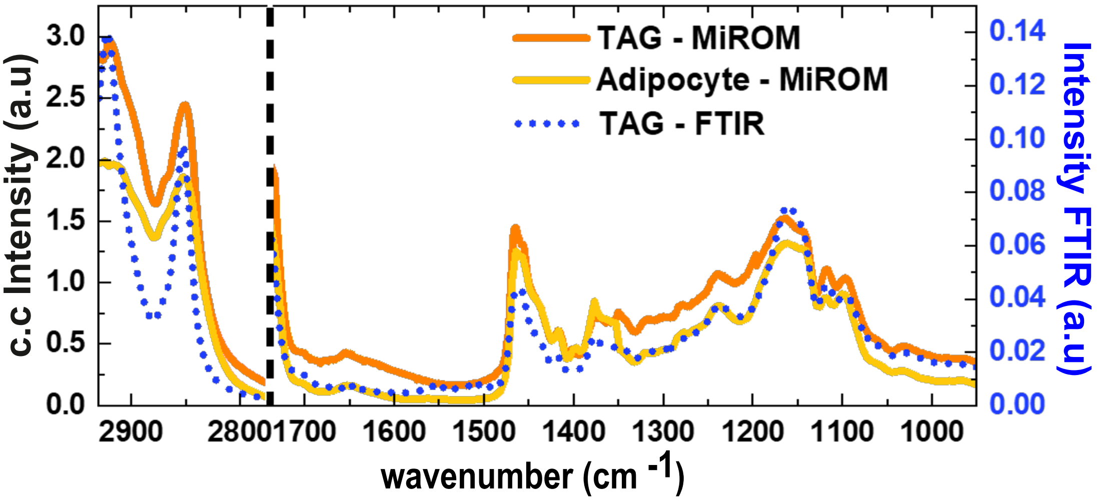

Supplementary Fig. 5: MiROM and FTIR spectra of adipocytes and pure triglycerides. MiROM spectrum of adipocyte (yellow) and pure triglycerides (orange) compared to ATR-FTIR spectroscopy (blue dotted line).

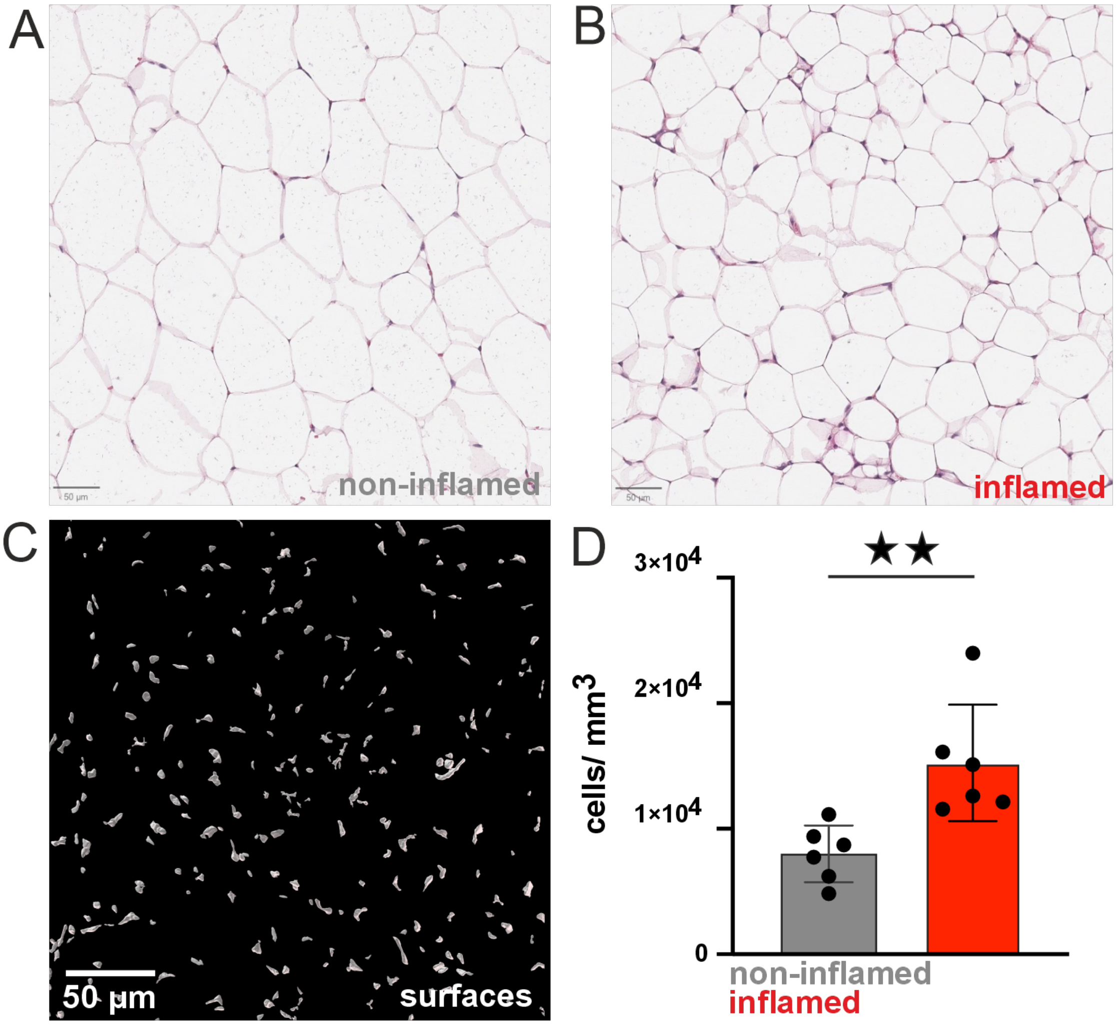

Supplementary Fig. 6: Histology and immunofluorescent imaging to demonstrate WAT inflammation. (A, B) Representative pictures of WAT sections of non-inflamed (A) or inflamed (B) WAT stained for H&E (scale bar 50 *µ*m). **(C, D)** infiltrating immune cells (macrophages) were labelled and surfaces used to quantify cell numbers in the non-inflamed and inflamed WAT (scale bar: 50 µm).

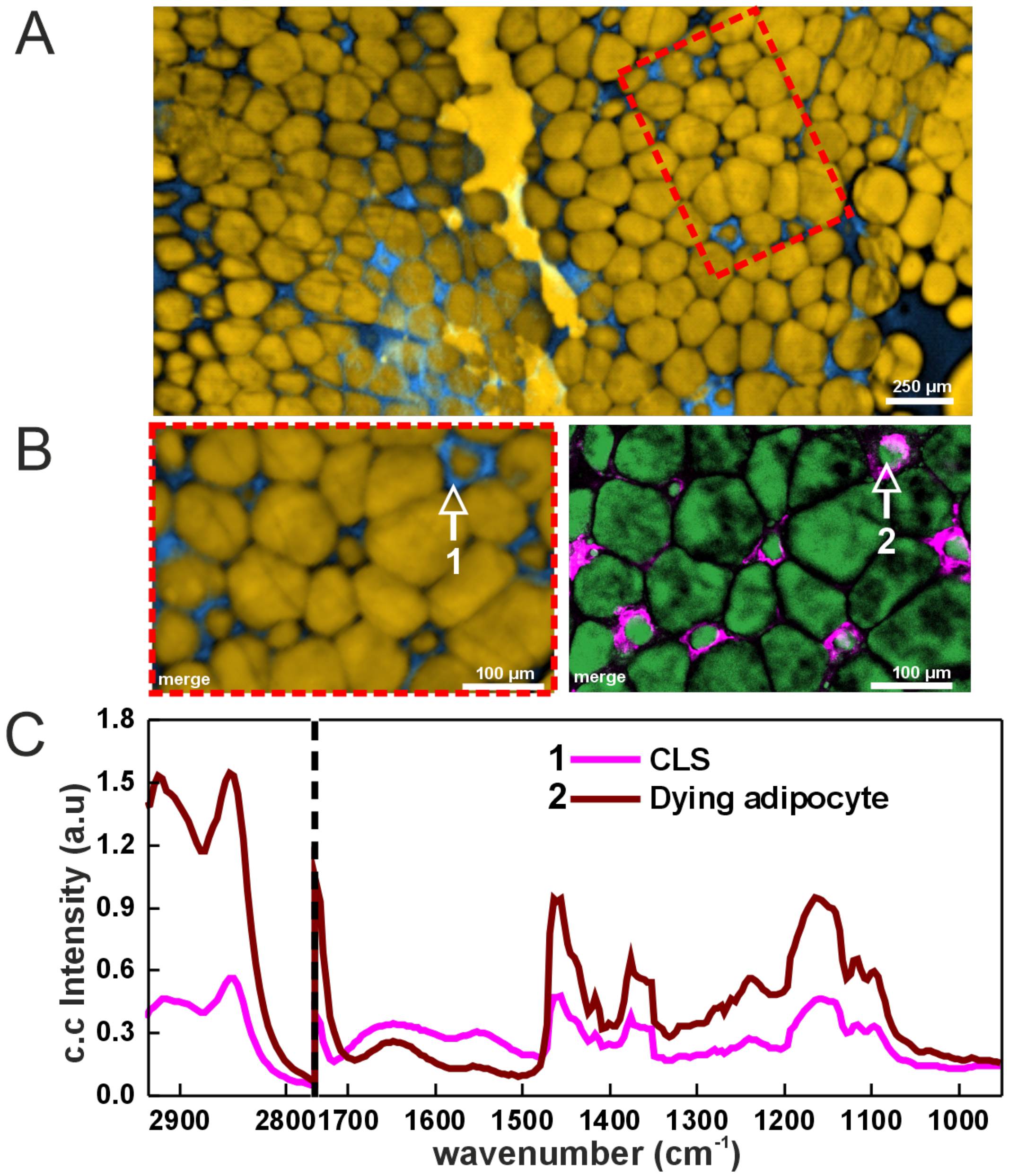

Supplementary Fig. 7: Complementary images and spectra to CLS shown in Fig. 2D-I.(**A**) Full FOV of inflamed tissue in **Fig. 2D-F** with several CLS shown in a merged 2852 and 1550 cm^{-1} micrograph. (**B**) The region of interest in **Fig. 2D-I** is taken from a cropped region from panel **A**, where two spectra are acquired in point 1 and 2. (**C**) MiROM spectra from CLS (point 1) and dying adipocyte (point 2).

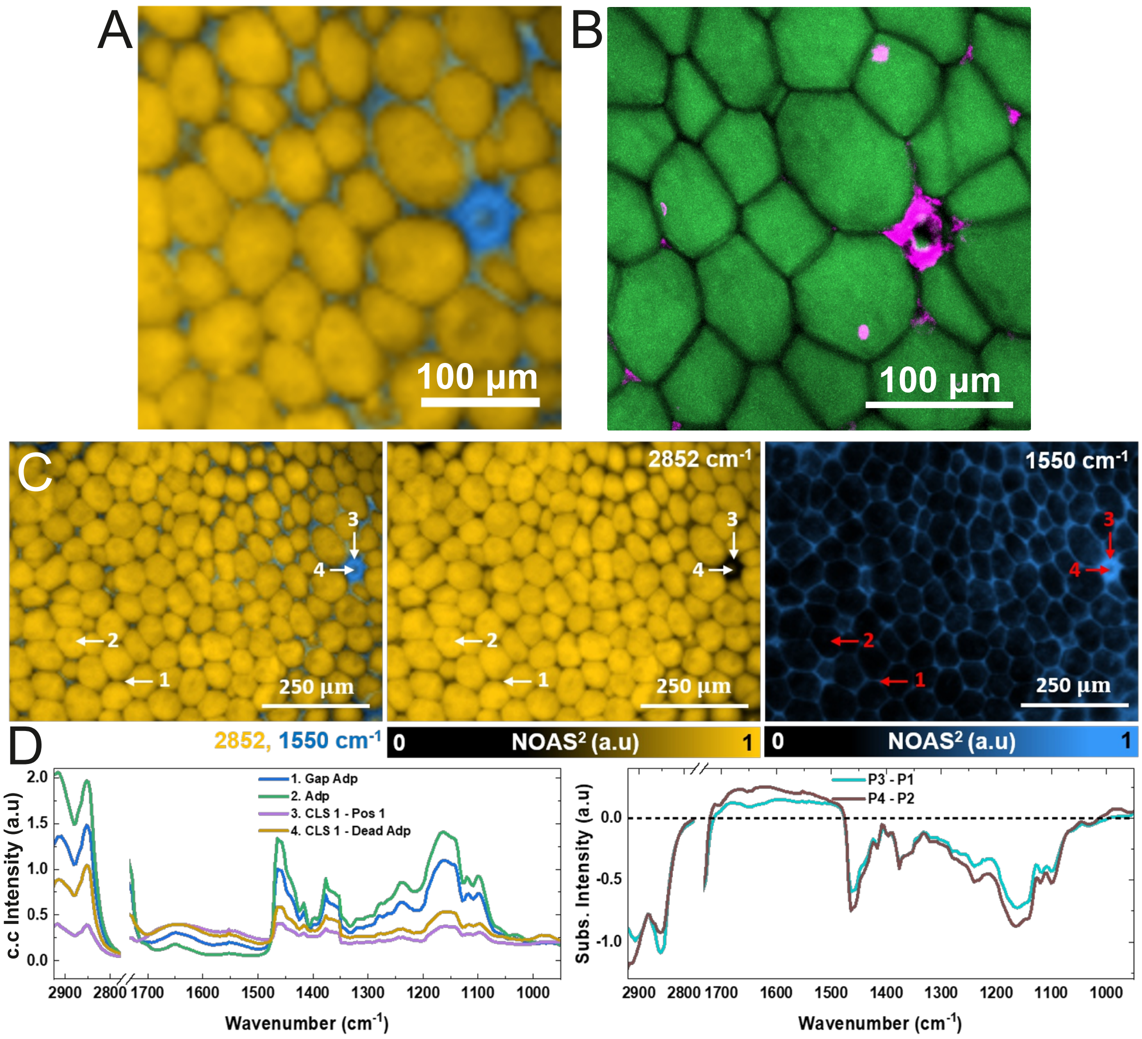

Supplementary Fig. 8: Spectra of CLS and adjacent features in WAT. (**A**) CLS and dying adipocyte are imaged with endogenous contrasts at 2852 and 1550 cm^{-1}, which matches with (**B**) LSCM micrograph of WAT labeled with BODIPY and MHC-II. (**C**) Selected points for spectral analysis: (1) Gap between adipocytes (1550 cm^{-1}), (2) Adipocyte (2852 cm^{-1}), (3) Macrophage accumulation (1550 cm^{-1}), and (4) dying adipocyte in the middle of CLS (2852 cm^{-1}). (**D**) We observe that the spectrum at macrophages accumulation (no. 3) produces a higher intensity in Amide I and II regions compared to gap between adipocytes (no. 1). Similarly, the dying adipocyte (no. 4) has a lower lipid concentration compared to an adjacent adipocyte (no. 2), reflected in a lower spectrum intensity in CH_2 region.

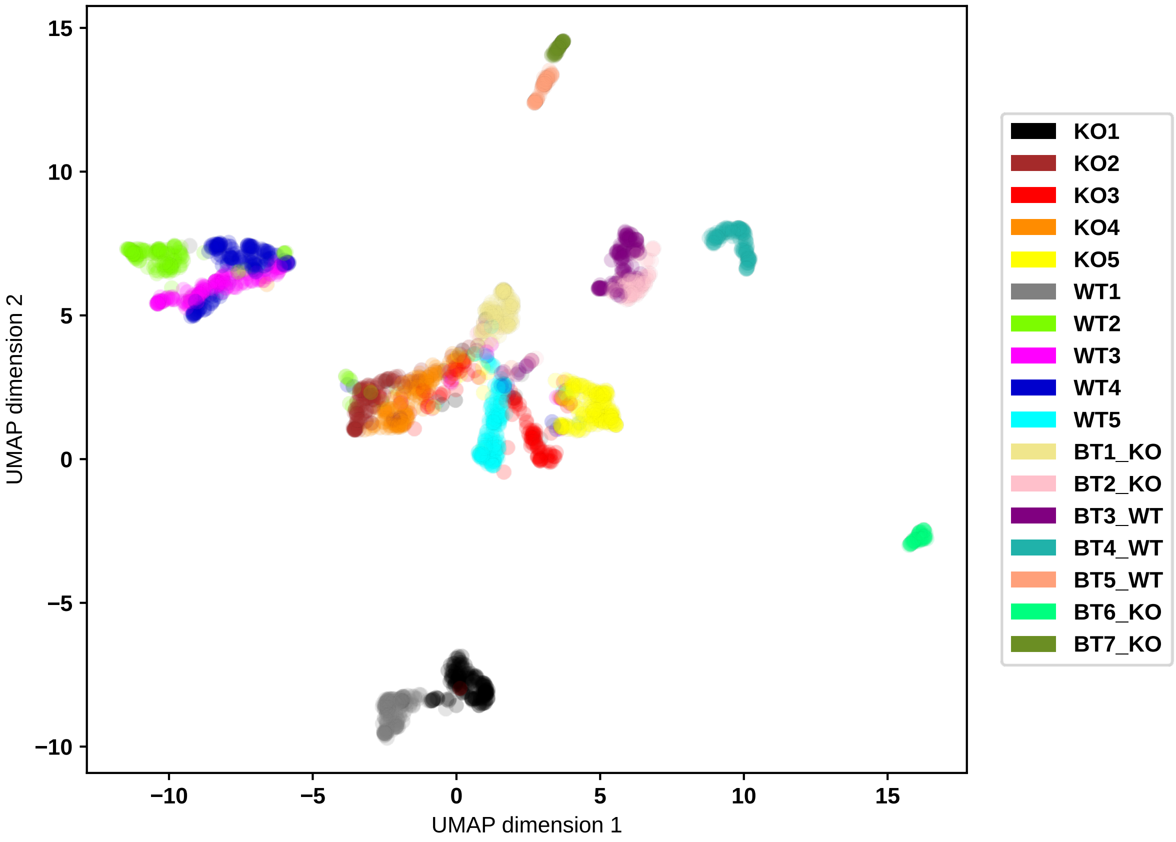

Supplementary Fig. 9: Spectral distribution from 17 samples represented in UMAP. Two-dimensional UMAP embedding of the adipocyte spectra from 17 mice samples. The clustering of the spectra shows effects beside the inflammation influence the spectra. For example, inflamed-1 (KO1) and non-inflamed1 (WT1) are isolated from other samples because the measurements were performed in a similar period of time. However, the differences between inflamed and non-inflamed tissues within this sample still can be seen from the separation of adipocyte populations between respective tissue samples.

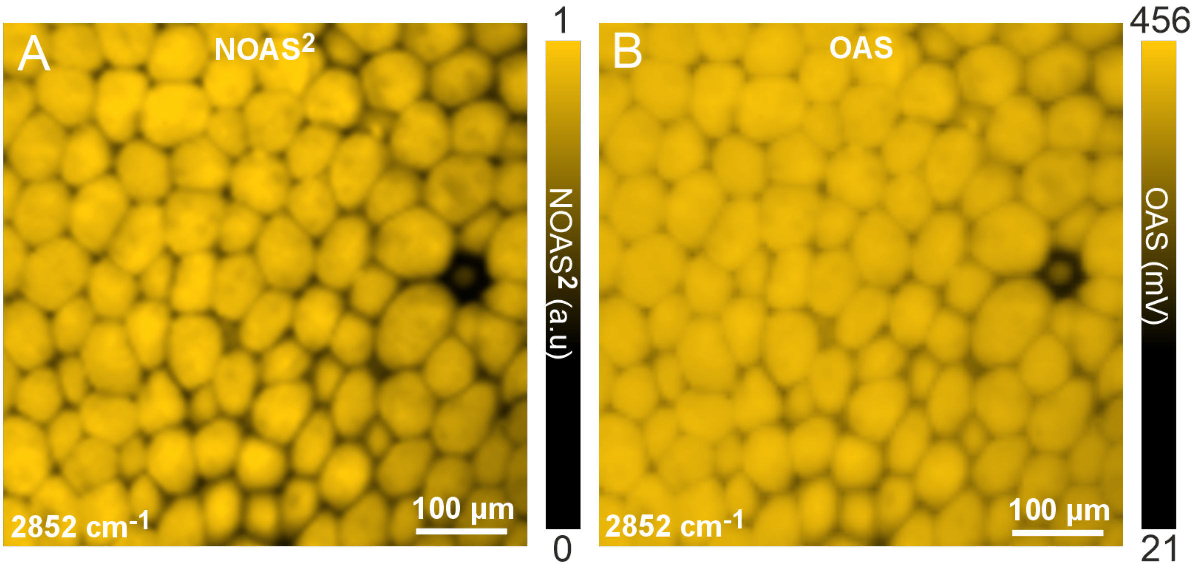

Supplementary Fig. 10: Comparison between normalized squared and raw optoacoustic micrograph. (A, B) Side by side comparison of **(A)** normalized squared and **(B)** raw OAS micrograph at 2852 cm^{-1} of **Fig. 1D**, showing a higher detail in normalized squared image due to squared contrast effect between dark and bright region.
